# Association between Triglyceride-Glucose Index and Prognosis in Critically Ill Patients with Acute Coronary Syndrome: Evidence from the MIMIC Database

**DOI:** 10.7150/ijms.107976

**Published:** 2025-02-26

**Authors:** Manqing Chen, Yuhui Yang, Weiwei Hu, Lingmin Gong, Zhenli Liao, Yifan Fu, Xingyan Li, Hongman Feng, Fangyao Chen

**Affiliations:** 1 Department of Epidemiology and Biostatistics, School of Public Health, Xi'an Jiaotong University Health Science Center, China.; 2 Department of Radiology, First Affiliate Hospital of Xi'an Jiaotong University, China.

**Keywords:** TyG index, acute coronary syndrome, short-term mortality, long-term mortality, length of stay in hospital, critically ill patients

## Abstract

**Background:** This study aimed to investigate the association between triglyceride-glucose (TyG) index and prognosis in critically ill patients with acute coronary syndrome (ACS), exploring potential heterogeneity of the association among patient subgroups with different characteristics.

**Methods:** Records of patients with ACS were extracted from the MIMIC-IV database. The association between TyG index and mortality was analyzed using Cox proportional-hazard regression model, while potential non-linear associations were assessed using restricted cubic spline (RCS) regression. Meanwhile, linear regression model was used to explore the association between TyG index and length of stay in hospital or ICU. Subpopulation Treatment Effect Pattern Plot (STEPP) was utilized to explore the potential heterogeneous subgroups. Time-dependent Receiver Operating Characteristic (ROC) curve analyses were performed to compare the predictive ability of different Cox proportional-hazard regression models (with or without TyG index).

**Results:** A total of 849 patients were enrolled. Multivariate Cox regression analyses demonstrated that TyG index was significantly associated with 28-day mortality (HR:2.13 [95%CI: 1.23-3.68], *P*<0.01) and 365-day mortality (HR:1.65 [95%CI: 1.11-2.47], *P*<0.01). RCS regression analyses revealed an inverted U-shaped association between TyG index and 28-day mortality (*P* for non-linearity=0.027) and a linear association between TyG index and 365-day mortality (*P* for non-linearity =0.086). There were subgroups specified by age for 28-day mortality (*P* for interaction=0.04) and 365-day mortality (*P* for interaction<0.01), with a cut-off point of 70 years old obtained by STEPP. TyG index was associated with a higher risk of mortality in subgroups aged ≤ 70 years old. Time-dependent ROC curve suggested that TyG index could slightly improve the prediction of mortality. A higher TyG index was associated with longer time of stay in hospital (*β*: 1.79 [95%CI: 0.06-3.52], *P*=0.04).

**Conclusions:** A higher TyG index is associated with both short-term and long-term all-cause mortality in critically ill patients with ACS, especially in short-term all-cause mortality. TyG index is associated with higher mortality risk in patient subgroups aged ≤ 70 years old. A higher TyG index is associated with longer time of stay in hospital. TyG index may serve as a useful prognostic marker for patient management and strategic decision-making in clinical settings.

## Background

Acute coronary syndrome (ACS) is a critical manifestation of cardiovascular disease (CVD), contributing significantly to high mortality rates 1, 2. Critically ill ACS patients in ICU often have severe comorbidities such as chronic kidney disease, diabetes, and geriatric syndromes, which further increase mortality and worsen prognosis 3-7. However, there are few studies on prognosis of critically ill patients with ACS. It has been proved that insulin resistance (IR) is associated with increased mortality in cardiovascular disease 8. The triglyceride-glucose (TyG) index, due to its convenience and cost-effectiveness, has emerged as a practical alternative for assessing insulin resistance (IR) 9, 10. TyG index has been identified to be associated with the prognosis of various diseases, including CVD 11, stroke 12, acute kidney injury (AKI) 13. Besides, published studies have indicated that TyG index was associated with all-cause mortality in critically ill patients with chronic kidney disease 14, sepsis 15, heart failure 16, stroke 17 or in the general population 18. However, the association between TyG index and prognosis in critically ill patients with ACS remains unclear and requires further investigation.

Associations between exposures and health outcomes tend to be heterogeneous across populations. Thus, subgroup analyses are extremely necessary for observational association studies, because it enable more precise and tailored approaches to treatment 19. Clinicians can develop targeted therapies that are more effective for specific patient populations and improve prognosis.

Therefore, we aimed to investigate the association between TyG index and all-cause mortality in critically ill patients with ACS, including short-term and long-term all-cause mortality. And we also assessed potential non-linear associations. Meanwhile, we explored potential subgroups, which could ensure that we find all possible potential subgroups. Additionally, we explored the association between TyG index and length of stay in hospital or ICU.

## Methods

### Study population

This was a retrospective observational study conducted based on public database. Research data were obtained from the Medical Information Mart for Intensive Care-IV (MIMIC-IV-2.0) database 20. The MIMIC database is a large and freely accessible database that comprises de-identified health-related data from patients who were admitted to the critical care units of the Beth Israel Deaconess Medical Center 20. MIMIC-IV contains data from 2008-2019. The authors were granted access to the dataset (ID: 42273093).

The inclusion criteria was that patients diagnosed with ACS. Patients were extracted by International Classification of Diseases (ICD) Code, including version 9 and version 10. The exclusion criteria were as follows: (1) with multiple hospital stays, only the first admission to intensive care unit (ICU) or the first admission to hospital was extracted; (2) younger than 18 years old; (3) with missing triglyceride or glucose records; (4) patients did not enter ICU.

### Variable extraction

In this study, Navicat Premium software (version 16) through structured query language (SQL) was used to extract variables. For laboratory indicators, only measurements obtained within 24 hours of admission to the ICU were extracted. If there are multiple measurements within 24 hours of admission to the ICU, the average value of these measurements was calculated as the final value.

Variables with more than 40% missing values were excluded. For variables with less than 40% missing values, the missingness was imputed using the k-nearest neighbors (KNN) algorithm (with *k*=11). KNN algorithm is a non-parametric method that does not assume any specific distribution of the data, and KNN can handle both numerical and categorical data 21. Additionally, KNN algorithm uses weighted averaging, where closer neighbors have a greater influence on the imputed value, enhancing the accuracy of imputations 22. Furthermore, KNN algorithm is robust to outliers, as it considers multiple neighbors, reducing the impact of noisy data points 23. Based on the aforementioned reasons, we have chosen the KNN algorithm for imputation, aiming to minimize the impact of missing values on the results and enhance the robustness of the findings.

The included variables can be categorized into six classes: demographics, laboratory indicators, vital signs, comorbidities, severity scores, and medication.

Demographic factors included gender, age, race, height and weight.

Laboratory variables included triglyceride, glucose, white blood cell (WBC), red blood cell (RBC), Platelet, Hemoglobin, Hematocrit, glycated hemoglobin A 1c (HbA 1c), Sodium, Potassium, Calcium, Chloride, Bicarbonate, high-density lipoprotein (HDL), low-density lipoprotein (LDL), total cholesterol (TC), cardiac troponin T (TnT), creatine kinase isoenzyme MB (CKMB), serum creatinine (Scr), blood urea nitrogen (BUN), alanine aminotransferase (ALT), aspartate aminotransferase (AST), alkaline phosphatase (ALP), Bilirubin, creatinine, prothrombin time (PT), active partial thromboplastin time (PTT), international normalized ratio (INR).

Vital signs include systolic blood pressure (SBP), diastolic blood pressure (DBP), heart rate, respiratory rate, and temperature.

Comorbidities include type 2 diabetes mellitus (T2DM), hypertension, hyperlipidemia, congestive heart failure (CHF), peripheral vascular disease (PVD), cerebrovascular disease, renal disease, chronic pulmonary disease (CPD), charlson comorbidity index (CCI).

Severity scores include acute physiology score III (APSIII), simplified acute physiology score (SAPSII) and oxford acute severity of illness score (OASIS).

Medications include Angiotensin-Converting Enzyme Inhibitor / Angiotensin II Receptor Blocker (ACEI/ARB), β- blocker, calcium channel blockers (CCB), diuretics, insulin, statin, Anti-ACS drugs, aspirin, and warfarin.

TyG index was calculated according to the formula 24: ln [fasting triglycerides (mg/dL) × fasting glucose (mg/dL)]/2.

Glomerular Filtration Rate (GFR) was calculated according to the formula proposed by Chronic Kidney Disease Epidemiology Collaboration (CKD-EPI) in 2009 25.

### Primary outcome and secondary outcomes

The primary outcomes of the study were 28-day and 365-day all-cause mortality. The secondary outcomes were 90-day, 180-day and 5-year all-cause mortality; length of stay in hospital and length of stay in ICU. The follow-up period started from the time of ICU admission.

### Statistical analyses

Patients were divided into two groups depending on whether they survived within 28 days or 365 days, respectively. The differences between two groups were evaluated using different methods depending on the data distribution. For continuous variables, a student *t*-test or a nonparametric test were used. For categorical variables, Pearson chi-square test or Fisher's exact test was utilized.

Kaplan-Meier (K-M) curves were used to compare survival outcomes between different TyG level groups stratified by quartile.

To explore the association between TyG index and mortality, univariable and multivariate Cox proportional-hazard regression models were used. To explore the association between TyG index and length of stay in hospital or ICU, linear regression models were utilized. TyG index was incorporated in these models as quartile or continuous variables.

Restricted cubic spline (RCS) regression was used to explore potential non-linear association between TyG index levels and mortality. Harrell suggests that setting the number of knots to four optimizes model fit, as it balances the smoothness of the curve while avoiding precision loss due to overfitting 26. Consistent with this, Harrell and other researchers recommend positioning the four knots of the RCS at the 5th, 35th, 65th, and 95th percentiles 26-28.

Subgroup analyses were performed to further investigate potential heterogeneous association between TyG index levels and mortality. We employed the Subpopulation Treatment Effect Pattern Plot (STEPP) method to identify potential subgroups for continuous variables, including age and BMI. The fundamental principle of the STEPP method is to divide the study subjects into multiple partially overlapping subgroups based on continuous covariates, and then estimate the treatment effect within each subgroup 29. This approach enables researchers to more easily identify subgroups that benefit more or less from the treatment, as it shows the results when each point is chosen as a subgroup split 29. Unlike empirical subgroup division, STEPP allows for a more comprehensive exploration of all potential subgroups that may exist 29.

Meanwhile, Cox proportional-hazard regression model and RCS regression analyses were also conducted in subgroups. Notably, TyG index was reclassified based on the nodes of the RCS in subgroup analyses.

Time-dependent receiver operating characteristic (ROC) curve 30 analyses were then performed to compare the predictive ability of different Cox proportional-hazard regression models (with or without TyG index). And the area under the ROC curve (AUC) was calculated. Notably, TyG index is reclassified based on the nodes of the RCS.

Two-sided *P* value of less than 0.05 was considered statistically significant, and all statistical analyses were performed using the R language (R version 4.3.1).

## Results

### Baseline characteristics

**Figure 1:**
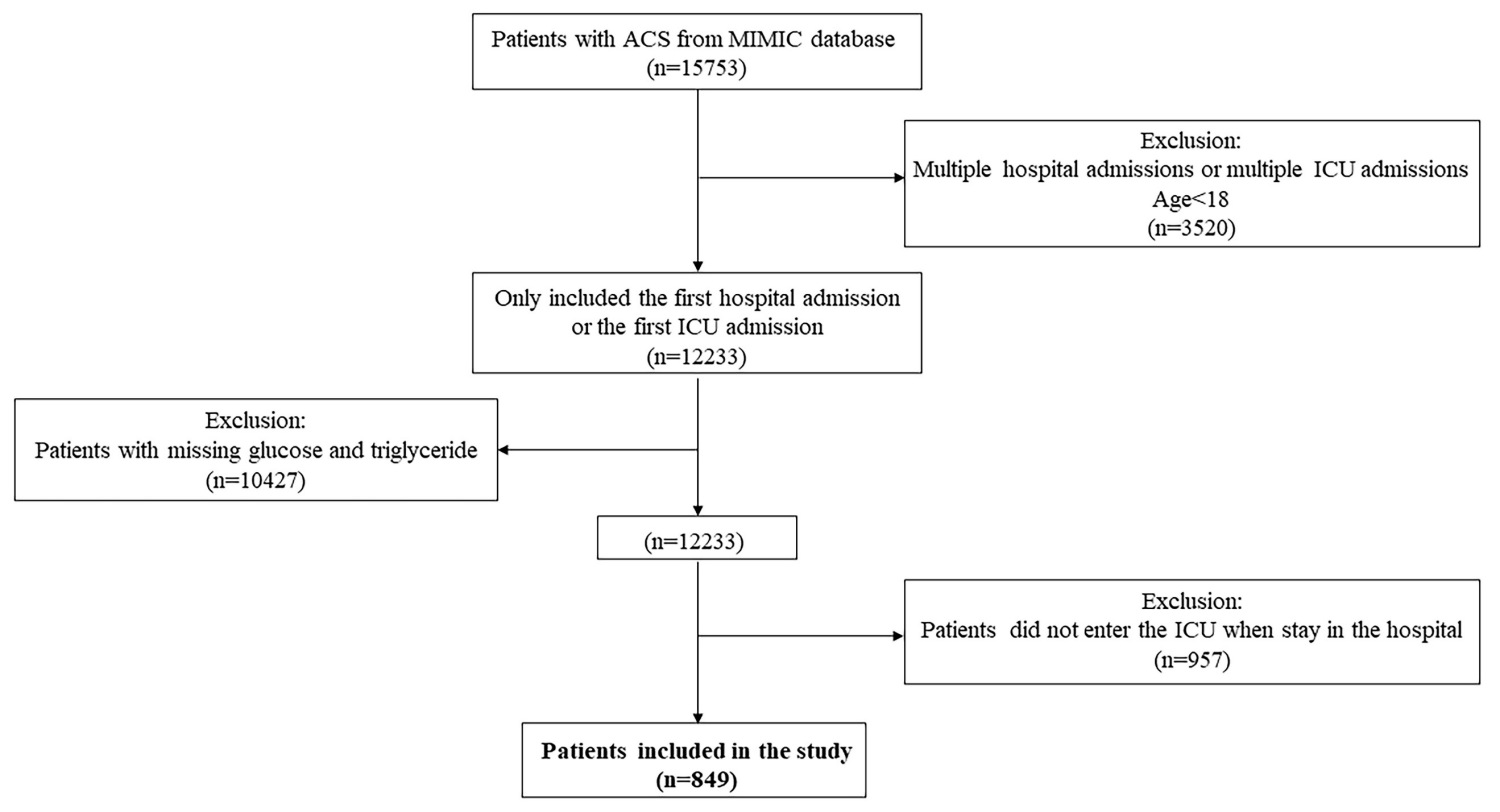
Flowchart of patient selection.

In this study, a total of 849 patients were enrolled. The detailed patient selection process was demonstrated in Fig. 1. The all-cause mortality of 28-day and 365-day were 15.19% and 26.03% respectively. And the all-cause mortality of 90-day, 180-day, and 5-year were 20.14%, 22.97% and 30.39%. The mean length of stay in hospital and ICU were 11 days and 4 days respectively. The quartiles of TyG index were Q1 (≤ 4.64), Q2 (> 4.64, ≤ 4.82), Q3 (> 4.82, ≤ 5.05) and Q4 (> 5.05) respectively.

In Table 1, a comparison of 28-day mortality between the survivors and non-survivors across the aforementioned variables was presented, including 720 survivors and 129 non-survivors. There was a statistically significant difference (*P=*0.04) between the two groups in TyG index as a continuous variable, and the TyG index of non-survivor group was higher. K-M curves (Supplementary Fig. S1-S2) illustrated the differences in survival outcomes of patients between TyG groups and mortality. However, no statistically significant differences were observed (28-day mortality: *P=*0.25; 365-day mortality: *P=*0.27). Similarly, there were no significant differences in K-M curves for 90-day mortality (*P=*0.30); 180-day mortality (*P=*0.23) and 5-year mortality. (*P=*0.64, Supplementary Figs. S3-S5).

In Table 1, Non-survivors tended to be older than the survivors. In addition, patients with T2DM, CHF, cerebrovascular disease and renal disease had a higher risk of 28-day mortality. Non-survivors demonstrated significantly higher levels of WBC, potassium, calcium, Scr, BUN, ALT, AST, PT, INR, HR and RR. Non-survivors had higher scores of CCI, OASIS, SAPSII and APSIII. The proportion of patients who received ACEI/ARB, β-blocker, statin, aspirin, warfarin, or Anti-ACS drugs during hospital was significantly lower among the non-survivors compared to the survivors.

### Association between TyG index and prognosis

As shown in Table 2, univariate Cox regression analyses showed that TyG index was associated with 28-day mortality when TyG index was incorporated as a continuous variable (HR:1.70 [95% CI: 1.04-2.78], *P*=0.04) or an ordinal variable (HR:1.67 [95%CI: 1.01-2.76], *P*=0.04).

The multivariate Cox regression model was used to assess the effect of exposure variables on the outcome measures, adjusting for covariates (Model1: adjusted for age, gender and BMI. Model2: adjusted for age, gender, BMI, hematocrit, potassium, calcium, LDL, ALT, ALP, PT, SBP, CCI, statin and Anti-ACS drugs.). Multivariate Cox regression analyses also showed that the association for both Model1 (HR: 2.26 [95% CI: 1.34-3.79], *P*<0.01) and Model2 (HR:1.62 [95% CI: 1.01-2.64], *P*=0.04) when TyG index was incorporated as a continuous variable. When TyG index was incorporated as an ordinal variable, the coefficients for TyG index were statistically significant for Model1 (Q4 vs. Q1, HR:2.14 [95%CI: 1.27-3.58], *P*<0.01) and Model2 (Q4 vs. Q1, HR:2.13 [95%CI: 1.23-3.68], *P*<0.01).

Meanwhile, the risk of 28-day mortality demonstrated an upward trend with increasing TyG index tertiles [Model2: Ref. vs. Q2:1.79(1.03-3.09,* P*=0.04) vs. Q3:2.13 (1.22-3.73,* P*<0.01) vs. Q4:2.13 (1.23-3.68,* P*<0.01)], with trend *P*-values below 0.05. The results showed that a higher TyG index was significantly associated with higher 28-day mortality.

In Model2, the association between TyG index and 365-day mortality was statistically significant when TyG index was incorporated as an ordinal variable (Q4 vs. Q1, HR:1.65 [95%CI: 1.11-2.47], *P*<0.01). However, when TyG index was a continuous variable, the association wasn't statistically significant (HR:1.44 [95%CI: 0.97-2.14], *P*=0.07). Interestingly, the value of hazard ratio for 28-day mortality was greater than that of 365-day mortality [2.13 (1.23-3.68, *P*<0.01) vs. 1.65 (1.11-2.47, *P*<0.01)] (Table 2).

The associations between TyG index and 90-day, 180-day and 5-year mortality were statistically significant when TyG index was incorporated as an ordinal variable in Model2 (Supplementary Table S1). Notably, when comparing the all-cause mortality across 90-day, 180-day and 5-year, it was found that hazard ratio of 90-day was greater than 180-day and hazard ratio of 180-day was greater than 5-year [90-day mortality: 1.85 (1.17-2.93, *P*<0.01) vs. 180-day mortality: 1.78 (1.16-2.74, *P*<0.01) vs. 5-year mortality: 1.47 (1.01-2.13, *P*=0.04)] (Supplementary Table S1).

As shown in Table 3, the association between TyG index and length of stay in hospital was statistically significant when TyG index was a continuous variable in Model2 (*β*: 1.79 [95%CI: 0.06-3.52], *P*=0.04). However, when the outcome was length of stay in ICU, the association wasn't statistically significant in Model2 (*β*: 0.84 [95%CI: -0.24-1.92], *P*=0.13).

**Figure 2:**
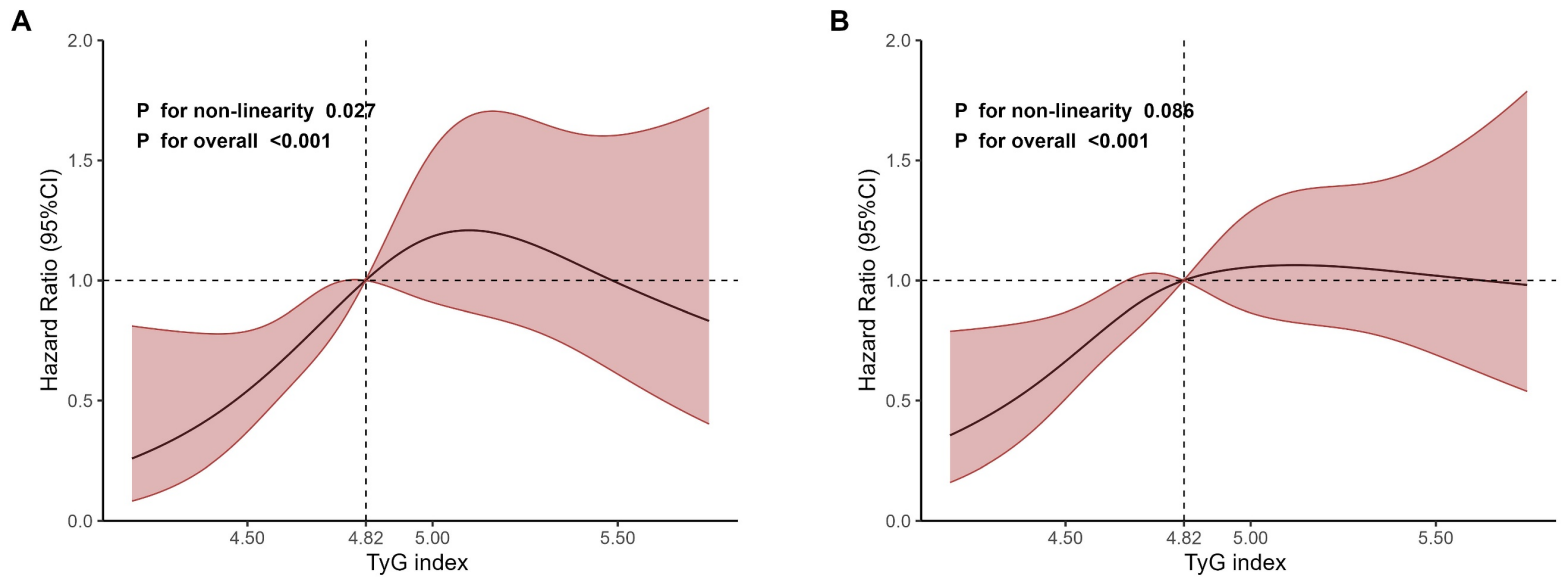
Restricted cubic spline regression analyses of TyG index for mortality. **A** 28-day mortality. **B** 365-day mortality.

As shown in Fig. 2, the RCS regression analyses revealed an inverted U-shaped association between TyG index and 28-day mortality, with 4.82 being the inflection point. The non-linearity was statistically significant (*P* for non-linearity=0.027, Fig. 2A). However, the non-linearity wasn't statistically significant for 365-day mortality (*P* for non-linearity=0.086, Fig. 2B).

Meanwhile, the RCS curve of TyG index obtained in multivariate Cox regression analyses for 90-day, 180-day and 5-year mortality showed an inverted U-shaped shape (Supplementary Fig. S6). However, none of these non-linearities were statistically significant (90-day mortality: *P*=0.249; 180-day mortality: *P*=0.133 and 5-year mortality: *P*=0.156).

### Subgroup analyses

**Figure 3:**
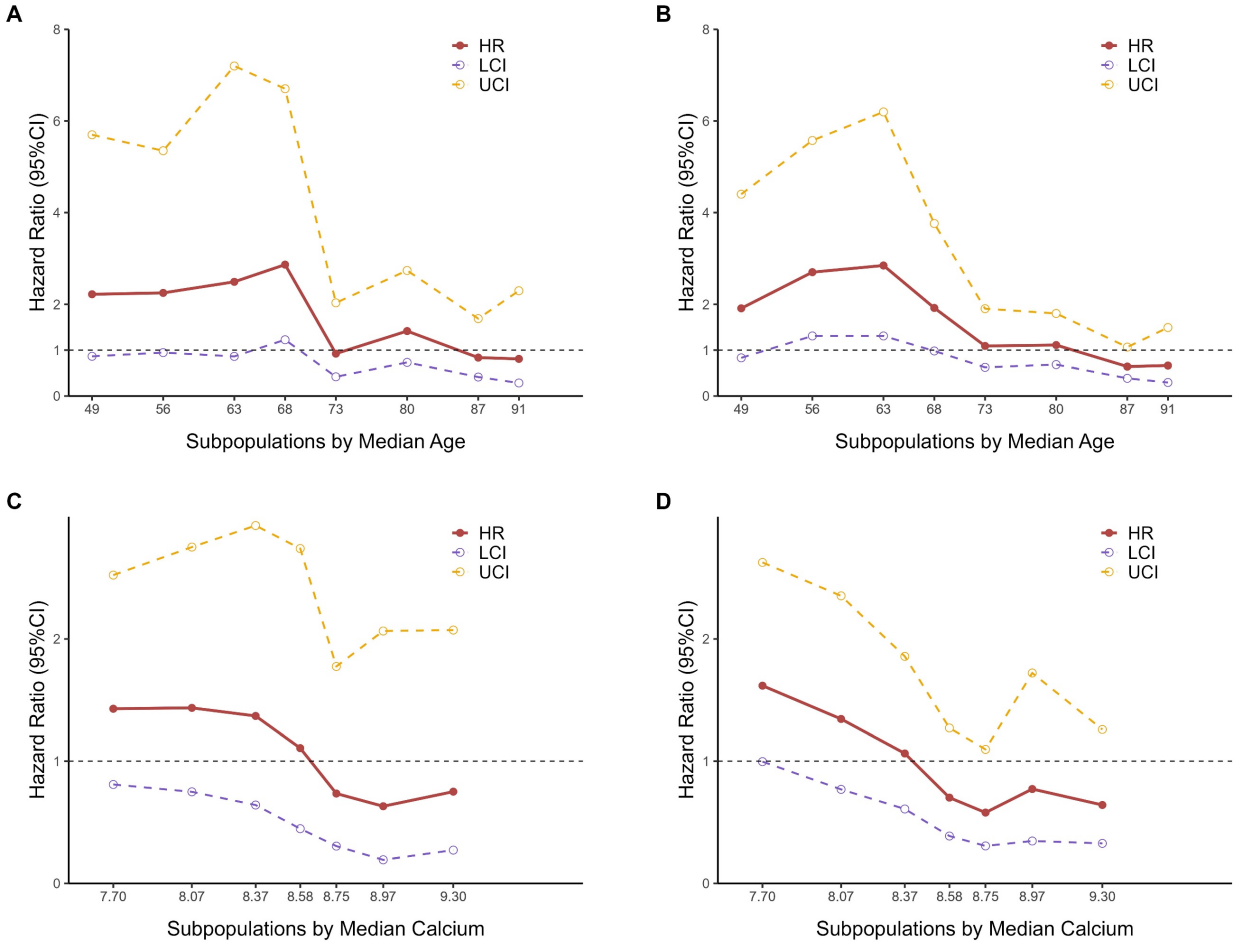
STEPP analyses of hazard ratios between two TyG index groups based on RCS nodes. **A** Subpopulations by median age for 28-day mortality. **B** Subpopulations by median age for 365-day mortality. **C** Subpopulations by median calcium levels for 28-day mortality. **D** Subpopulations by median calcium levels for 365-day mortality.

The results of the STEPP analyses for age indicated that the hazard ratios for 28-day mortality (Fig. 3A) and 365-day mortality (Fig. 3B) varied across different subpopulations. The hazard ratio between the two groups, as defined by RCS nodes, was higher among younger individuals. Notably, there was a marked decline in the hazard ratio around the median age, between 68 and 73 years old, suggesting the presence of heterogeneity in hazard ratios across different age groups. This observation led us to consider that a meaningful cut-off point likely exists within this age range.

We ultimately chose 70 years as the cutoff point for stratifying subpopulations by age, as it not only falls within the observed range but also offers practical advantages for clinical application. Choosing 70 as a round number simplifies interpretation and communication of results, and aligns well with common age stratifications used in clinical practice and previous studies, enhancing the comparability of our findings.

Similarly, according to the results of STEPP analyses for calcium levels (Fig. 3C-3D), BMI (Fig. S7A-S7B), and potassium levels (Supplementary Fig. S7C-S7D), we chose 8.6 mEq/L for calcium levels, 29 kg/m^2^ for BMI and 4.0 mEq/L for potassium levels as the cut-off point for subpopulations respectively.

**Figure 4:**
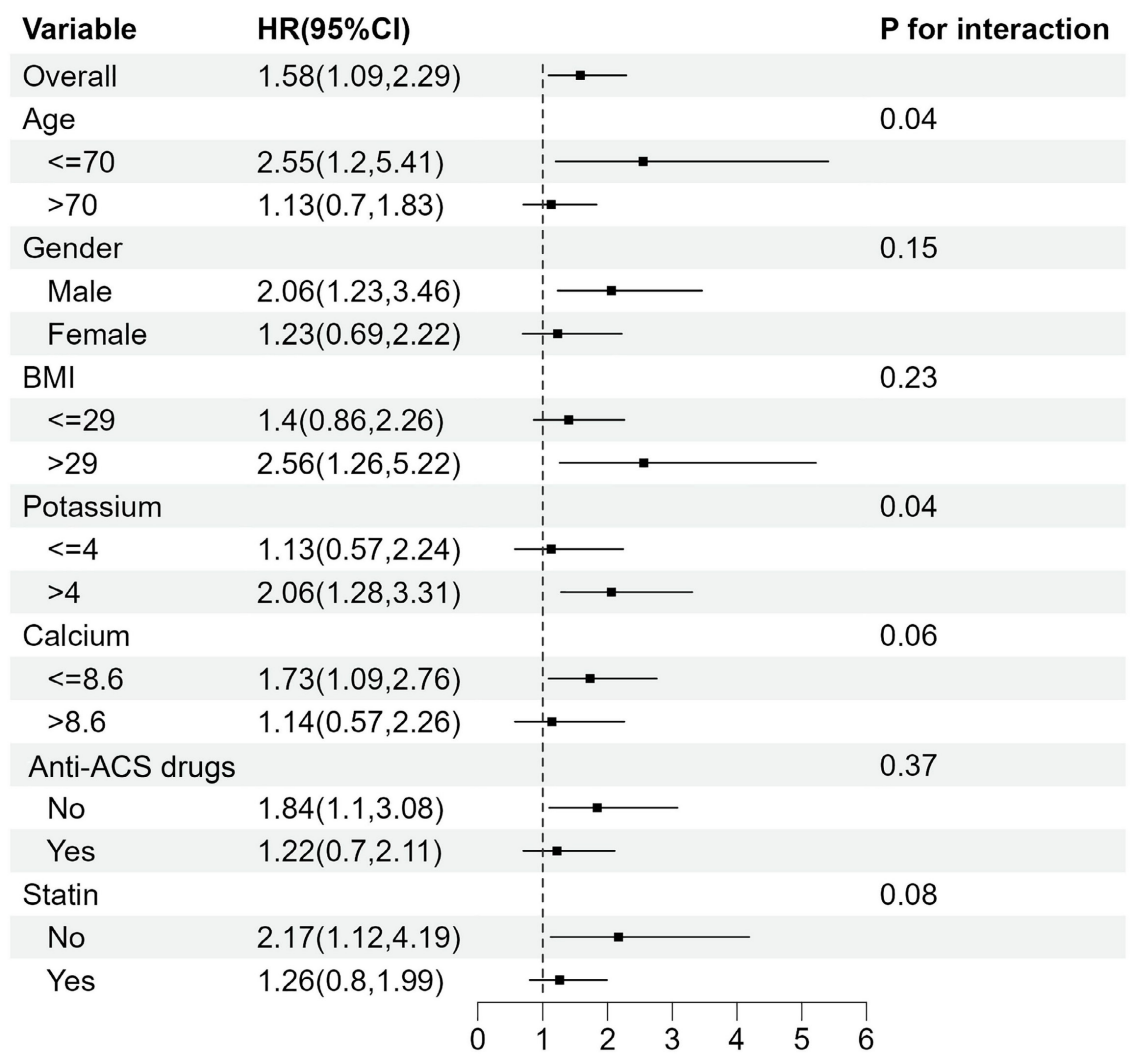
Subgroup analyses for the association between TyG index and 28-day mortality.

The association between TyG index and mortality was further examined in multiple subgroups, considering age, gender, BMI, potassium, calcium, Anti-ACS drugs and statin use. For 28-day mortality (Fig. 4), TyG index was significantly associated with a higher risk of mortality in subgroups aged <= 70 years old (HR 2.55 [95% CI: 1.20-5.41], *P* for interaction=0.04) and those with potassium levels > 4 mEq/L (HR 2.06 [95% CI: 1.28-3.31], *P* for interaction=0.04).

**Figure 5:**
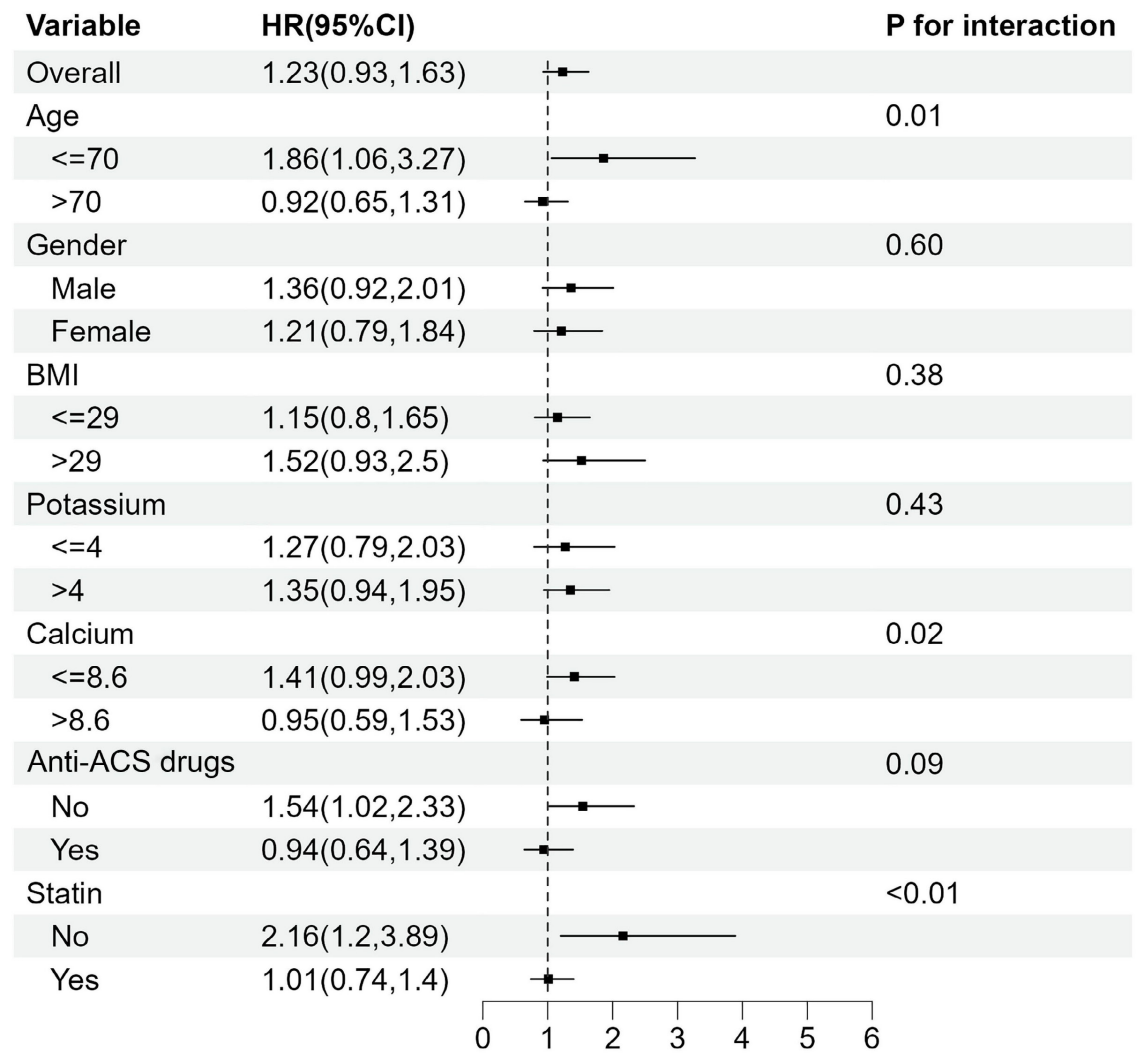
Subgroup analyses for the association between TyG index and 365-day mortality.

For 365-day mortality (Fig. 5), TyG index was significantly associated with a higher risk of mortality in subgroups aged ≤ 70 years old (HR 1.86 [95% CI: 1.06-3.27], *P* for interaction=0.01) and those who didn't take statin (HR 2.16 [95% CI: 1.20-3.89], *P* for interaction<0.01). Meanwhile, the interaction in subgroups with different calcium levels was statistically significant (*P* for interaction=0.02).

The patient subgroup aged ≤ 70 years old always had a higher risk for 90-day mortality (*P* for interaction=0.01, Supplementary Fig. S8), 180-day mortality (*P* for interaction=0.01, Supplementary Fig. S9) and 5-year mortality (*P* for interaction<0.01, Supplementary Fig. S10).

**Figure 6:**
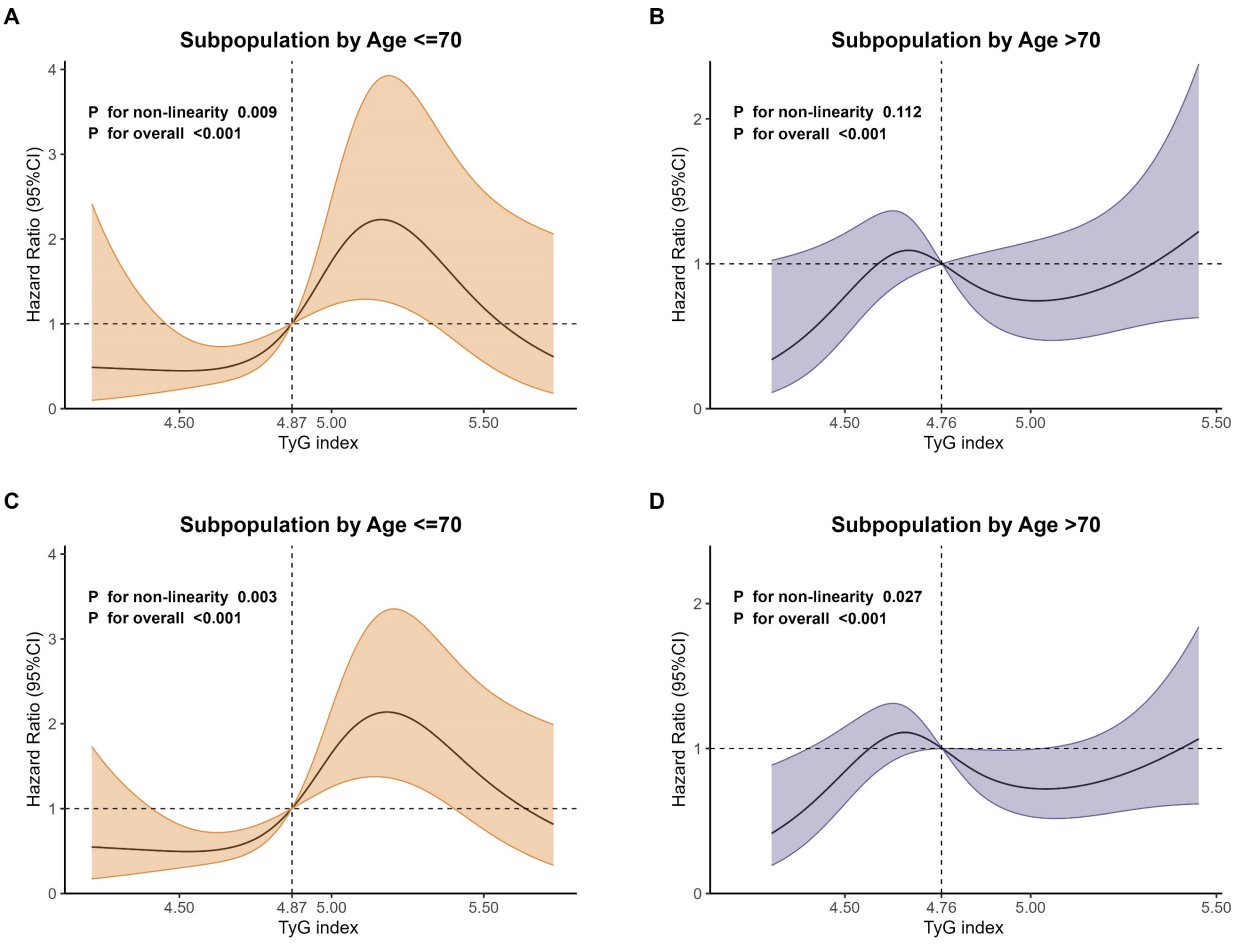
RCS curves of TyG index for mortality in age subgroups. **A** Subpopulation by age ≤ 70 years old for 28-day mortality. **B** Subpopulation by age >70 years old for 28-day mortality.** C** Subpopulation by age ≤ 70 years old for 365-day mortality. **D** Subpopulation by age >70 years old for 365-day mortality.

As shown in Fig. 6, the RCS regression analyses were performed in age-defined subpopulations to assess the association between the TyG index and 28-day as well as 365-day mortality. The results showed that, for 28-day mortality, the non-linearity was statistically significant (*P* for non-linearity=0.009) in subpopulations aged ≤ 70 years old (Fig. 6A). However, the non-linearity wasn't statistically significant (*P* for non-linearity=0.112) in subpopulations aged > 70 years old (Fig. 6B).

For 365-day mortality, the non-linearity was statistically significant in subpopulations aged ≤ 70 years old (*P* for non-linearity=0.003, Fig. 6C) and > 70 years old (*P* for non-linearity=0.027, Fig. 6D), but with different patterns.

## Discussion

To the best of our knowledge, this study was the first to examine the association of TyG index for both short-term and long-term all-cause mortality in critically ill patients with ACS. Meanwhile, this study investigated the association between TyG index and length of stay in hospital or ICU in critically ill patients with ACS. We also employed the STEPP method to explore potential subgroups and conducted subgroup analyses.

We observed that TyG index was significantly associated with 28-day mortality, 365-day mortality and length of stay in hospital in critically ill patients with ACS after adjusting for confounders. This association always holds when TyG index is incorporated in the models as quartile or continuous variables. Meanwhile, we observed an inverted U-shaped association between TyG index and 28-day mortality and a linear association between TyG index and 365-day mortality.

For the subgroup analyses, the age cut-off of 70 years old was determined using the STEPP method. Based on this, we found the TyG index was associated with a higher risk of mortality in patients aged ≤ 70 years for both 28-day and 365-day mortality.

TyG index slightly improved the predictive performance of the basic risk model for all-cause mortality (Supplementary Fig. S11-S12), and it appears to be a promising index for prevention and risk stratification in critically ill patients with ACS.

### Association between TyG index and prognosis

Our study found that TyG index was associated with all-cause mortality of critically ill patients with ACS, which was consistent with the conclusions of previous studies conducted based on ACS patients with non-critical conditions. Wang and Yang's study showed evidence from CCC-ACS (Improving Cardiovascular Care in China-ACS) database that after adjusting for confounders, patients in TyG index quartile group 3 or 4 showed increased risks of major adverse cardiovascular events (MACE), and nearly half of MACE was all-cause mortality in hospital (short-term mortality) 31. Chen *et al.* also found that TyG index was associated with a higher risk of MACEs in ACS patients, and over one-third of MACE was all-cause long-term mortality 32. Shen *et al.* found that TyG index was associated with all-cause long-term mortality in the oldest-old patients with ACS and diabetes mellitus 33. Several other studies have presented similar conclusions that TyG index was associated with mortality in ACS patients with non-critical conditions 34-36. However, most of these previous studies primarily focused on the general populations with ACS and this association has not been validated in critically ill patients with ACS. Our results confirmed that this association also existed in critically ill patients with ACS, providing significant assistance in risk stratification for the occurrence of death in critically ill ACS patients.

Additionally, these previous studies focused only on the association with either short-term or long-term mortality. Our study found that TyG index was associated with both short-term and long-term all-cause mortality in critically ill patients with ACS. Our findings suggested that critically ill patients with a high TyG index require additional attention and appropriate medical measures, as they may have a higher mortality. For critically ill patients with ACS who have a high TyG index, it is essential to maintain close and ongoing monitoring of their condition, even if no death occurs in the short term. This includes enhancing follow-up visits and conducting relevant examinations, as the risk of long-term mortality remains high.

Few studies have compared the prognostic value of the TyG index for short-term versus long-term all-cause mortality. Wang *et al.* 37 found that the hazard ratio of the TyG index for 30-day all-cause mortality was higher than that for 365-day all-cause mortality, concluding that the TyG index had a stronger predictive ability for short-term mortality in critically ill patients with coronary artery disease (CAD). In this study, we were the first to compare the association between the TyG index and both short-term and long-term all-cause mortality in critically ill patients with ACS. We found that the hazard ratio for short-term mortality was greater than that for long-term mortality.

The association between the TyG index and long-term mortality is weaker than that with short-term mortality in critically ill patients with ACS. This phenomenon may be related to several factors. Firstly, critically ill patients with ACS experience significant stress responses during the acute phase, which can lead to increased levels of the TyG index. A high TyG index may increase the risk of complications such as cardiogenic shock, heart failure, and acute kidney injury. These stress responses have a considerable impact on patient's health in the short term, making the association between the TyG index and short-term mortality stronger. Secondly, treatment interventions can significantly improve patients' conditions in the short term, but their impact on long-term prognosis may be smaller. Therefore, the predictive value of the TyG index in the short term may be higher. Furthermore, long-term prognosis is influenced by various factors, including patients' overall health status, lifestyle, and management of chronic diseases. The combined effect of these factors may weaken the association between the TyG index and long-term mortality.

Based on our findings, we concluded that TyG index yielded higher prognostic value for short-term than long-term all-cause mortality in critically ill patients with ACS. The importance of this finding lies in the fact that short-term and long-term mortality in critically ill patients have distinct predictive factors and intervention windows. Therefore, accurately assessing the role of the TyG index in both contexts is crucial for developing individualized treatment strategies. Particularly in the management of critically ill ACS patients, from acute care to long-term recovery, integrating such biomarkers can significantly improve prognosis evaluation and optimize treatment strategies.

Meanwhile, we found that a higher TyG index was significantly associated with longer time of stay in hospital in critically ill patients with ACS. Several previous studies have also found the association between higher TyG index and longer time of stay in hospital for patients with diseases other than ACS 38, 39. Fang and Zhang have confirmed the association between TyG index and length of stay in hospital in patients with sepsis 39 and in patients with heart failure and type 2 diabetes 38, respectively. Our results confirmed this association in critically ill patients with ACS, which was similar to the findings of previous studies. This finding provides valuable insights into the importance of monitoring patients with a high TyG index during hospitalization. Comprehensive and integrated management may reduce the length of stay in hospital for patients with a high TyG index, which can help reduce patients' suffering and optimize healthcare resource allocation.

The possible mechanisms of the association between TyG index and mortality are as follows. IR can disrupt glucose metabolism, leading to chronic hyperglycemia. This condition triggers oxidative stress and causes an inflammatory response, resulting in cell damage, which further impacts cardiovascular outcomes 40, 41. TyG index, which serves as a surrogate marker for IR, may affect cardiovascular outcomes by increasing the complexity of coronary artery lesions 32. Thus, it further affected the mortality and the prognosis of patients.

### Subgroup analyses

This study further examined the heterogeneity of mortality risk across various subgroups. Using the STEPP methodology, we found that patients aged ≤ 70 years old and those with calcium levels ≤ 8.6 mEq/L exhibited a higher risk associated with the TyG index and mortality. It is important to note that the boundaries explored with STEPP were not fixed values but fluctuated around these thresholds. Subgroup analyses based on this boundary are the most appropriate, as they were selected after testing all possible boundaries, rather than relying solely on prior experience.

Our subgroup analyses indicated that critically ill patients aged ≤ 70 years old with ACS who had a higher TyG index were at greater risk of mortality. This finding aligns with the results of Zhang *et al.* 42, Cai *et al.* 17, Zheng *et al.* 15, and Huang *et al.* 43, but contradicts the findings of Shen *et al.* 33. This may be because our patients were critically ill, and older patients tend to have more comorbidities. The increase in TyG index showed a minimal association with higher mortality in critically ill patients aged > 70 years old with ACS, which means TyG index may have less prognostic value in these patients. Clinically, when TyG index increases in patients aged ≤70 years old, we need to pay extra attention and give more timely clinical measures to reduce the mortality of patients.

We also found that TyG index had a higher risk in patients with calcium levels < 8.6 mEq/L. Previous studies have demonstrated that calcium and IR can influence each other based on pathophysiological and metabolic mechanisms and calcium was inversely associated with IR 44. Published research has already suggested that Calcium and TyG index were associated with CAD 45 and hypertension 46. We hypothesize that there may be a mutual or bi-directional influence between calcium and TyG index for all-cause mortality in critically ill patients with ACS, but it requires further validation.

### Strengths and limitations

This study demonstrates the prognostic value of the TyG index in critically ill patients with ACS, offering insights that can aid clinical decision-making. Sensitivity analyses, conducted after adjusting for confounders, further confirmed the robustness of our findings. This association remained consistent whether the TyG index was included in the models as quartile categories or as a continuous variable, ensuring the reliability of the results. TyG index, an easily measurable marker of insulin resistance, is effective in predicting mortality and hospital length of stay in this patient population.

Moreover, we employed the STEPP analyses to identify potential subgroups, providing a more nuanced understanding of high-risk populations. The results of these subgroup analyses could assist in recognizing high-risk patients, guiding therapeutic decisions, and improving outcomes in critically ill ACS patients. Our results indicate that an elevated TyG index is a useful marker for identifying patients with aged ≤ 70 years old at high risk of mortality in critically ill patients with ACS.

However, several limitations of the present study should be noted. Notably, The Global Registry of Acute Coronary Events (GRACE) score has been the best discriminative index as a predictor of prognosis in ACS patients and is recommended for risk assessment. However, the present study did not compare the ability of TyG index and GRACE score due to the unavailability of data on GRACE score in MIMIC database. Notably, previous studies have shown that TyG index has equal prognostic value with GRACE score 47-49.

Secondly, our analyses were retrospective and based on observational data, which makes it difficult to establish causal associations. Thirdly, the calculation of TyG index requires fasting triglyceride and glucose indices, but we are not sure if they were measured under fasting, which makes it more uncertain for the results. Fourthly, due to the limitations of MIMIC databases, we could only get TyG index at baseline, and the changes in TyG index during the follow-up period could not be included. Then, data about the causes of hospitalization was missing in MIMIC database, so we could not have information on whether the patient was in hospital for ACS. Besides, the study's sample size is moderate and need larger-scale cohort studies to reinforce our conclusions. Future prospective studies are needed to address these limitations and offer a more in-depth understanding of the TyG index in predicting prognosis for critically ill patients with ACS.

## Conclusions

A higher TyG index is associated with both short-term and long-term all-cause mortality in critically ill patients with ACS, especially in short-term all-cause mortality. TyG index is associated with higher mortality risk in patient subgroups aged ≤ 70 years old. A higher TyG index is associated with longer time of stay in hospital. Assessing the TyG index enhances the overall disease evaluation and supports the development of more effective, personalized treatment and management strategies. When TyG index increases in critically ill patients with ACS, particularly those aged ≤ 70 years old, it is crucial to provide timely and effective treatment to prevent disease progression, which may help decrease the time of ICU stays and mortality rates. TyG index may serve as a useful prognostic marker for patient management and strategic decision-making in clinical settings.

## Supplementary Material

Supplementary figures.

## Figures and Tables

**Table 1:** Baseline characteristics between survivors and non-survivors for 28-day mortality

	Overall (N=849)	Survivor (N=720)	Non‑survivor (N=129)	*P* value
**Demographics**				
Age (years)	67.6 ± 14.3	66.7 ± 14.2	72.8 ± 14.0	<0.001
Gender, n (%)				
Male	529 (62.3%)	455 (63.2%)	74 (57.4%)	0.246
Female	320 (37.7%)	265 (36.8%)	55 (42.6%)	
Race, n (%)				
Black	513 (60.4%)	447 (62.1%)	66 (51.2%)	
White	58 (6.8%)	46 (6.4%)	12 (9.3%)	
Asian	24 (2.8%)	21 (2.9%)	3 (2.3%)	
Hispanic/Latino	17 (2%)	15 (2.1%)	2 (1.6%)	
Other	37 (4.4%)	35 (4.9%)	2 (1.6%)	
Unknown	200 (23.6%)	156 (21.7%)	44 (34.1%)	0.018
Height (m)	170.0 ± 8.7	170.0 ± 8.7	170.0 ± 8.8	0.954
Weight (kg)	84.3 ± 22.0	84.6 ± 21.8	82.9 ± 23.5	0.428
BMI (kg/m^2^)	29.1 ± 5.6	29.1 ± 5.3	28.7 ± 6.7	0.485
**Laboratory parameters**				
Tyg index	4.9 ± 0.3	4.8 ± 0.3	4.9 ± 0.3	0.045
Tyg index (quantile)				
Q1	213 (25.1%)	188 (26.1%)	25 (19.4%)	0.297
Q2	212 (25%)	180 (25%)	32 (24.8%)	
Q3	212 (25%)	179 (24.9%)	33 (25.6%)	
Q4	212 (25%)	173 (24%)	39 (30.2%)	
White blood cell (K/μL)	12.3 ± 5.4	11.8 ± 4.8	14.8 ± 7.4	<0.001
Red blood cell (m/μL)	4.0 ± 0.7	4.0 ± 0.7	3.7 ± 0.8	<0.001
Platelet (K/μL)	222.0 ± 86.3	223.1 ± 84.0	215.6 ± 98.0	0.411
Hemoglobin (g/dL)	11.9 ± 2.1	12.1 ± 2.0	11.1 ± 2.3	<0.001
Hematocrit (%)	35.8 ± 5.9	36.1 ± 5.6	34.0 ± 6.7	0.001
Glycated hemoglobin A 1c (%)	6.2 ± 1.2	6.2 ± 1.3	6.1 ± 1.1	0.330
Sodium (mEq/L)	138.3 ± 4.2	138.3 ± 3.9	138.8 ± 5.5	0.303
Potassium (mEq/L)	4.2 ± 0.5	4.2 ± 0.5	4.5 ± 0.7	<0.001
Calcium (mEq/L)	8.5 ± 0.6	8.6 ± 0.6	8.3 ± 0.8	<0.001
Chloride (mEq/L)	103.1 ± 5.2	103.2 ± 4.9	102.7 ± 6.8	0.413
Bicarbonate (mEq/L)	22.9 ± 3.9	23.4 ± 3.7	20.5 ± 4.4	<0.001
High-density lipoprotein (mg/dL)	45.5 ± 15.5	46.0 ± 15.5	42.9 ± 15.3	0.035
Low-density lipoprotein (mg/dL)	91.7 ± 39.6	94.1 ± 39.7	78.0 ± 36.0	<0.001
Total cholesterol (mg/dL)	161.9 ± 47.0	165.4 ± 46.4	142.1 ± 45.5	<0.001
Cardiac troponin T (ng/mL)	2.9 ± 4.0	2.9 ± 3.7	3.0 ± 5.5	0.926
Creatine kinase isoenzyme MB (mg/dL)	84.9 ± 101.8	88.6 ± 101.5	64.2 ± 101.8	0.012
Serum creatinine (mg/dL)	1.4 ± 1.3	1.3 ± 1.2	2.0 ± 1.6	<0.001
Blood urea nitrogen (mg/dL)	25.3 ± 18.3	23.3 ± 16.2	36.5 ± 24.3	<0.001
GFR (mL/min/1.73m^2^)	76.8 ± 34.3	81.0 ± 33.1	53.0 ± 31.3	<0.001
Alanine aminotransferase (IU/L)	120.4 ± 388.9	89.0 ± 282.0	295.8 ± 720.2	0.002
Aspartate aminotransferase (IU/L)	277.6 ± 879.0	212.6 ± 696.0	640.5 ± 1497.0	0.002
Alkaline phosphatase (IU/L)	82.3 ± 57.6	79.4 ± 55.2	98.7 ± 67.5	0.003
Bilirubin (mg/dL)	0.9 ± 2.0	0.8 ± 1.9	1.3 ± 2.8	0.064
				
Prothrombin Time (second)	14.3 ± 4.4	13.9 ± 3.7	16.6 ± 6.7	<0.001
Active partial thromboplastin time (second)	48.5 ± 24.9	48.5 ± 24.6	48.3 ± 26.8	0.944
International normalized ratio	1.3 ± 0.5	1.3 ± 0.4	1.6 ± 0.9	<0.001
**Vital signs**				
Systolic blood pressure (mmHg)	118.0 ± 16.5	118.4 ± 15.7	115.5 ± 20.5	0.131
Diastolic blood pressure (mmHg)	66.4 ± 11.5	66.9 ± 11.3	63.5 ± 12.1	0.002
Heart rate (beats/minute)	80.7 ± 14.9	79.7 ± 14.3	86.2 ± 17.2	<0.001
Respiratory rate (beats/minute)	19.5 ± 3.5	19.2 ± 3.2	21.6 ± 4.1	<0.001
Temperature (℃)	36.8 ± 0.6	36.8 ± 0.5	36.8 ± 0.8	0.759
**Comorbidities**				
Type 2 diabetes mellitus, n (%)				
No	629 (74.1%)	543 (75.4%)	86 (66.7%)	0.048
Yes	220 (25.9%)	177 (24.6%)	43 (33.3%)	
Hypertension, n (%)				
No	259 (30.5%)	226 (31.4%)	33 (25.6%)	0.224
Yes	590 (69.5%)	494 (68.6%)	96 (74.4%)	
Hyperlipidemia, n (%)				
No	396 (46.6%)	338 (46.9%)	58 (45%)	0.749
Yes	453 (53.4%)	382 (53.1%)	71 (55%)	
Congestive heart failure, n (%)				
No	438 (51.6%)	385 (53.5%)	53 (41.1%)	0.013
Yes	411 (48.4%)	335 (46.5%)	76 (58.9%)	
Peripheral vascular disease, n (%)				
No	762 (89.8%)	644 (89.4%)	118 (91.5%)	0.588
Yes	87 (10.2%)	76 (10.6%)	11 (8.5%)	
Cerebrovascular disease, n (%)				
No	698 (82.2%)	617 (85.7%)	81 (62.8%)	<0.001
Yes	151 (17.8%)	103 (14.3%)	48 (37.2%)	
Renal disease, n (%)				
No	681 (80.2%)	589 (81.8%)	92 (71.3%)	0.008
Yes	168 (19.8%)	131 (18.2%)	37 (28.7%)	
Chronic pulmonary disease, n (%)				
No	670 (78.9%)	576 (80%)	94 (72.9%)	0.087
Yes	179 (21.1%)	144 (20%)	35 (27.1%)	
Charlson comorbidity index	6.4 ± 2.8	6.1 ± 2.6	8.0 ± 3.0	<0.001
**Severity scores**				
Oxford acute severity of illness score	31.4 ± 9.7	29.7 ± 8.9	40.9 ± 8.8	<0.001
Simplified acute physiology score II	33.8 ± 13.8	31.2 ± 12.0	48.2 ± 14.5	<0.001
Acute physiology score III	44.1 ± 24.9	39.4 ± 21.1	70.2 ± 28.5	<0.001
**Medication**				
Angiotensin-Converting Enzyme Inhibitor / Angiotensin II Receptor Blocker, n (%)				
No	326 (38.4%)	213 (29.6%)	113 (87.6%)	<0.001
Yes	523 (61.6%)	507 (70.4%)	16 (12.4%)	
*β*-blocker, n (%)				
No	138 (16.3%)	76 (10.6%)	62 (48.1%)	<0.001
Yes	711 (83.7%)	644 (89.4%)	67 (51.9%)	
Calcium channel blockers, n (%)				
No	715 (84.2%)	605 (84%)	110 (85.3%)	0.821
Yes	134 (15.8%)	115 (16%)	19 (14.7%)	
Diuretic, n (%)				
No	410 (48.3%)	358 (49.7%)	52 (40.3%)	0.061
Yes	439 (51.7%)	362 (50.3%)	77 (59.7%)	
Insulin, n (%)				
No	402 (47.3%)	373 (51.8%)	29 (22.5%)	<0.001
Yes	447 (52.7%)	347 (48.2%)	100 (77.5%)	
Statin, n (%)				
No	102 (12%)	58 (8.1%)	44 (34.1%)	<0.001
Yes	747 (88%)	662 (91.9%)	85 (65.9%)	
Aspirin, n (%)				
No	61 (7.2%)	34 (4.7%)	27 (20.9%)	<0.001
Yes	788 (92.8%)	686 (95.3%)	102 (79.1%)	
Warfarin, n (%)				
No	657 (77.4%)	538 (74.7%)	119 (92.2%)	<0.001
Yes	192 (22.6%)	182 (25.3%)	10 (7.8%)	
Anti-ACS drugs, n (%)				
No	270 (31.8%)	202 (28.1%)	68 (52.7%)	<0.001
Yes	579 (68.2%)	518 (71.9%)	61 (47.3%)	

**Table 2:** Association between TyG index and mortality (Cox regression)

Outcome	Groups	Non-adjusted Model	Model1	Model2
28-day mortality	Continuous	**1.70(1.04,2.78)**	**2.26(1.34,3.79)**	**1.62(1.01,2.64)**
	≤ 4.64	Ref	Ref	Ref
	(4.64, 4.82)	1.33(0.79,2.24)	1.41(0.84,2.39)	**1.79(1.03,3.09)**
	(4.82, 5.05)	1.36(0.81,2.28)	1.55(0.92,2.62)	**2.13(1.22,3.73)**
	> 5.05	**1.67(1.01,2.76)**	**2.14(1.27,3.58)**	**2.13(1.23,3.68)**
365-day mortality	Continuous	1.35(0.92,1.98)	**1.93(1.28,2.89)**	1.44(0.97,2.14)
	≤ 4.64	Ref	Ref	Ref
	(4.64, 4.82)	1.23(0.85,1.80)	1.32(0.91,1.93)	**1.55(1.05,2.30)**
	(4.82, 5.05)	1.01(0.68,1.49)	1.18(0.79,1.75)	1.42(0.93,2.15)
	> 5.05	1.36(0.94,1.97)	**1.84(1.25,2.69)**	**1.65(1.11,2.47)**

Bold values denote statistical significance at the *P* < 0.05 level Model 1: Adjusted for age, gender and BMI Model 2: Adjusted for age, gender, BMI, hematocrit, potassium, calcium, low-density lipoprotein, alanine aminotransferase, alkaline phosphatase, prothrombin time, systolic blood pressure, Charlson comorbidity index, statin and anti-ACS drugs

**Table 3:** Association between TyG index and length of stay (Linear regression)

Outcome	Groups	Non-adjusted Model	Model1	Model2
Length of hospital stay	Continuous	**2.50(0.71,4.29)**	**2.98(1.15,4.81)**	**1.79(0.06,3.52)**
	≤ 4.64	Ref	Ref	Ref
	(4.64, 4.82)	0.55(-1.16,2.26)	0.60(-1.11,2.31)	0.81(-0.78,2.4)
	(4.82, 5.05)	1.05(-0.66,2.76)	1.29(-0.43,3.01)	1.01(-0.59,2.59)
	> 5.05	1.62(-0.09,3.33)	2.01(0.26,3.75)	1.01(-0.63,2.66)
Length of ICU stay	Continuous	**1.30(0.22,2.37)**	**1.32(0.21,2.42)**	0.84(-0.24,1.92)
	≤ 4.64	Ref	Ref	Ref
	(4.64, 4.82)	-0.28(-1.3,0.76)	-0.28(-1.31,0.75)	-0.31(-1.31,0.68)
	(4.82, 5.05)	0.54(-0.49,1.57)	0.54(-0.49,1.58)	0.36(-0.63,1.36)
	> 5.05	0.67(-0.36,1.70)	0.69(-0.37,1.74)	0.22(-0.81,1.25)

Bold values denote statistical significance at the *P* < 0.05 level Model 1: Adjusted for age, gender and BMI Model 2: Adjusted for age, gender, BMI, hematocrit, potassium, calcium, low-density lipoprotein, alanine aminotransferase, alkaline phosphatase, prothrombin time, systolic blood pressure, charlson comorbidity index, statin and Anti-ACS drugs
