# Supplementary material for: Association between Triglyceride-Glucose Index and Prognosis in Critically Ill Patients with Acute Coronary Syndrome: Evidence from the MIMIC Database
Source: Int J Med Sci. 2025 Feb 26;22(7):1528–41. doi: 10.7150/ijms.107976 (PMC11905271; doi:10.7150/ijms.107976)
Supplement: Supplementary file 1 — Supplementary figures. [file ijmsv22p1528s1.pdf]

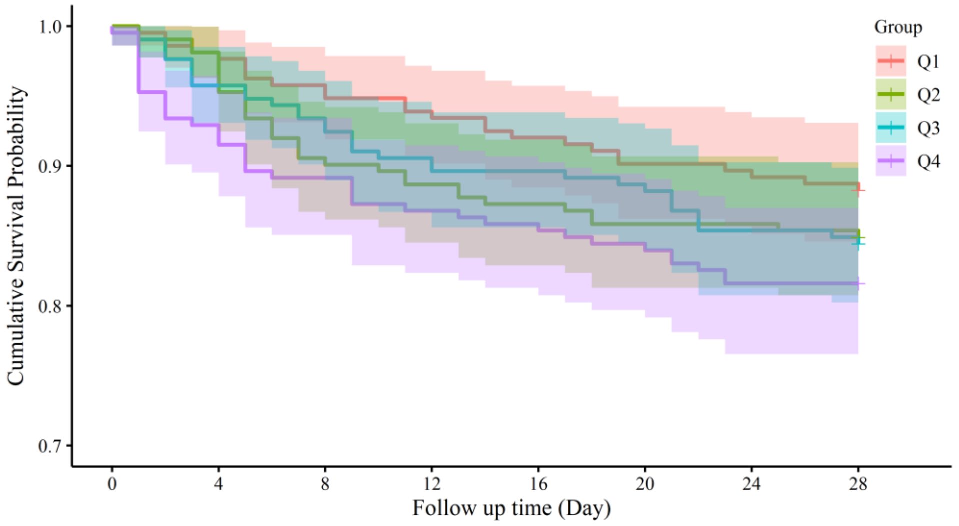

Number at risk

Q1	213	209	204	200	196	192	191	189
Q2	212	208	192	188	185	182	182	181
Q3	212	203	198	192	190	188	181	180
Q4	212	197	189	184	182	179	173	173

Fig. S1 Kaplan–Meier survival analyses curves for 28-day mortality.

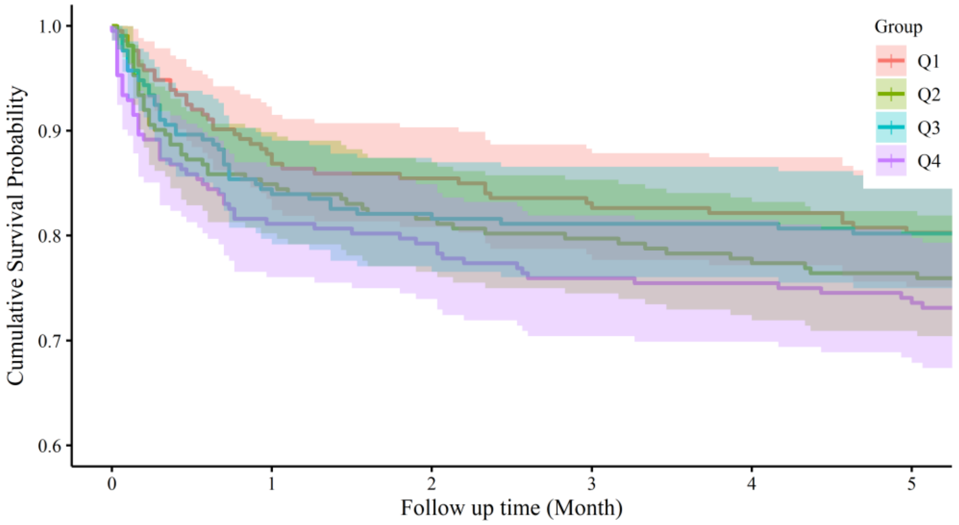

Number at risk

Q1	213	187	182	177	175	171
Q2	212	180	173	169	165	162
Q3	212	179	174	172	172	170
Q4	212	172	168	161	160	157

Fig. S2 Kaplan–Meier survival analyses curves for 5-year mortality.

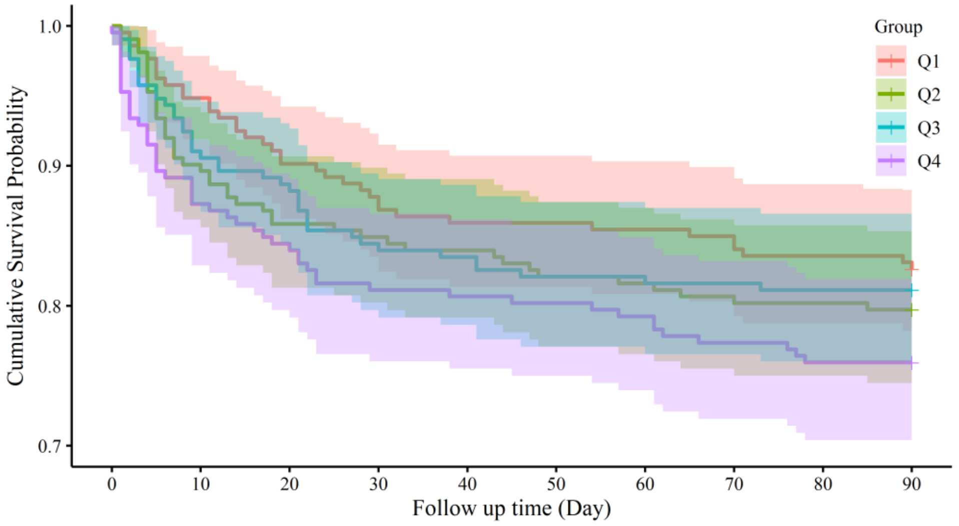

Number at risk

Q1	213	202	192	187	183	183	182	181	178	177
Q2	212	191	182	180	178	174	173	171	170	169
Q3	212	193	188	179	177	174	174	173	172	172
Q4	212	185	179	172	171	170	168	164	161	161

Fig. S3 Kaplan–Meier survival analyses curves for 90-day mortality.

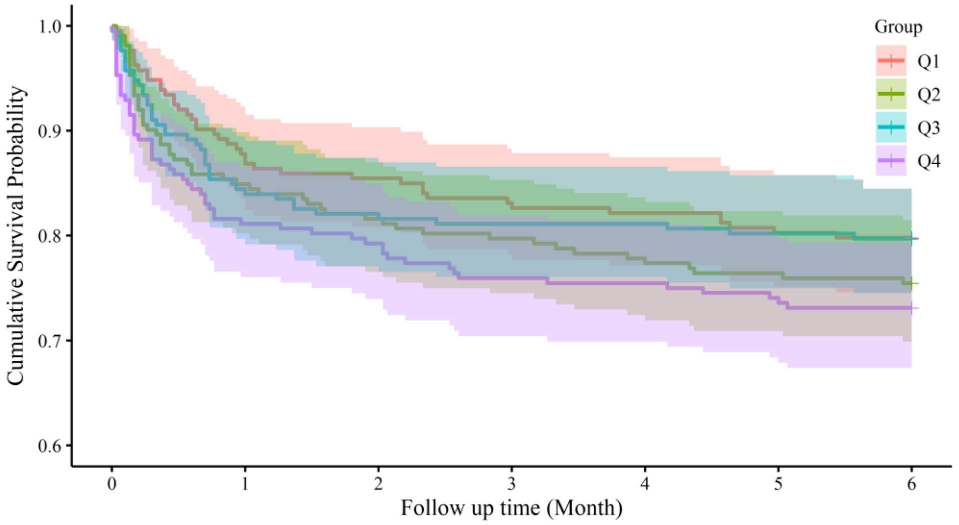

Number at risk

Q1	213	187	182	177	175	171	170
Q2	212	180	173	169	165	162	160
Q3	212	179	174	172	172	170	169
Q4	212	172	168	161	160	157	155

Fig. S4 Kaplan–Meier survival analyses curves for 180-day mortality.

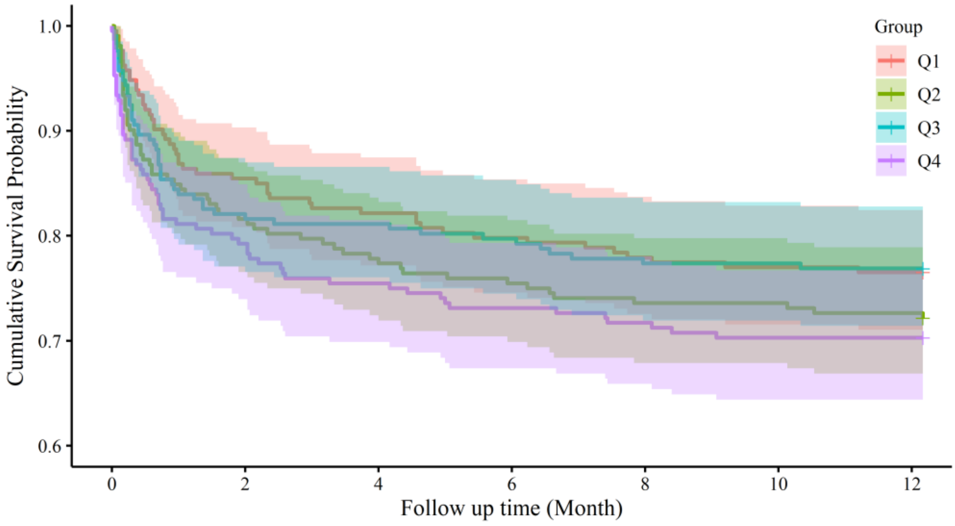

Number at risk

Q1	213	182	175	170	166	164	163
Q2	212	173	165	160	156	156	154
Q3	212	174	172	169	164	164	163
Q4	212	168	160	155	152	149	149

Fig. S5 Kaplan–Meier survival analyses curves for 365-day mortality.

Table S1 Association of TyG index with mortality (Cox regression)

Outcome	Groups	Non-adjusted Model		Model1		Model2	
		HR (95% CI)	***P* value**	HR (95% CI)	***P* value**	HR (95% CI)	***P* value**
90-day mortality	Continuous	1.550(1.006,2.389)	**0.0467**	2.132(1.353,3.361)	**0.0011**	1.611(1.045,2.484)	**0.0308**
	Q1 (≤ 4.64; N =213)	Ref	Ref	Ref	Ref	Ref	Ref
	Q2 (> 4.64, ≤4.82; N =212)	1.208(0.778,1.874)	0.3999	1.290(0.830,2.006)	0.2570	1.540(0.975,2.430)	0.0639
	Q3 (> 4.82, ≤5.05; N =212)	1.116(0.714,1.745)	0.6309	1.286(0.819,2.020)	0.2748	1.629(1.012,2.623)	**0.0445**
	Q4 (> 5.05; N =212)	1.481(0.970,2.261)	0.0691	1.935(1.250,2.995)	**0.0031**	1.853(1.171,2.933)	**0.0084**
	P for trend	/	0.1020	/	**0.0050**	/	**0.0107**
180-day mortality	Continuous	1.428(0.950,2.148)	0.0869	2.001(1.300,3.078)	**0.0016**	1.510(1.001,2.280)	**0.0495**
	Q1 (≤ 4.64; N =213)	Ref	Ref	Ref	Ref	Ref	Ref
	Q2 (> 4.64, ≤4.82; N =212)	1.260(0.841,1.887)	0.2621	1.354(0.903,2.031)	0.1424	1.646(1.079,2.510)	**0.0208**
	Q3 (> 4.82, ≤5.05; N =212)	1.030(0.675,1.572)	0.8908	1.190(0.777,1.823)	0.4245	1.519(0.967,2.386)	0.0695
	Q4 (> 5.05; N =212)	1.428(0.961,2.121)	0.0780	1.897(1.261,2.854)	**0.0021**	1.784(1.160,2.744)	**0.0084**
	P for trend	/	0.1660	/	**0.0066**	/	**0.0187**
5-year mortality	Continuous	1.248(0.872,1.786)	0.2258	1.800(1.231,2.631)	**0.0024**	1.409(0.977,2.034)	0.0668
	Q1 (≤ 4.64; N =213)	Ref	Ref	Ref	Ref	Ref	Ref
	Q2 (> 4.64, ≤4.82; N =212)	1.128(0.799,1.594)	0.4937	1.210(0.855,1.710)	0.2819	1.420(0.992,2.031)	0.0551
	Q3 (> 4.82, ≤5.05; N =212)	0.989(0.694,1.411)	0.9521	1.150(0.804,1.644)	0.4452	1.348(0.926,1.963)	0.1188

Q4 (> 5.05; N =212)	1.198(0.850,1.688)	0.3016	1.625(1.141,2.314)	**0.0071**	1.470(1.014,2.131)	**0.0422**
P for trend	/	0.4539	/	**0.0151**	/	0.0659

Bold values denote statistical significance at the *P* < 0.05 level

Model 1: Adjusted for Age, Gender and BMI

Model 2: Adjusted for Age, Gender, BMI, Hematocrit, Potassium, Calcium, LDL, ALT, ALP, PT, SBP, CCI, Statin and Anti-ACS drugs

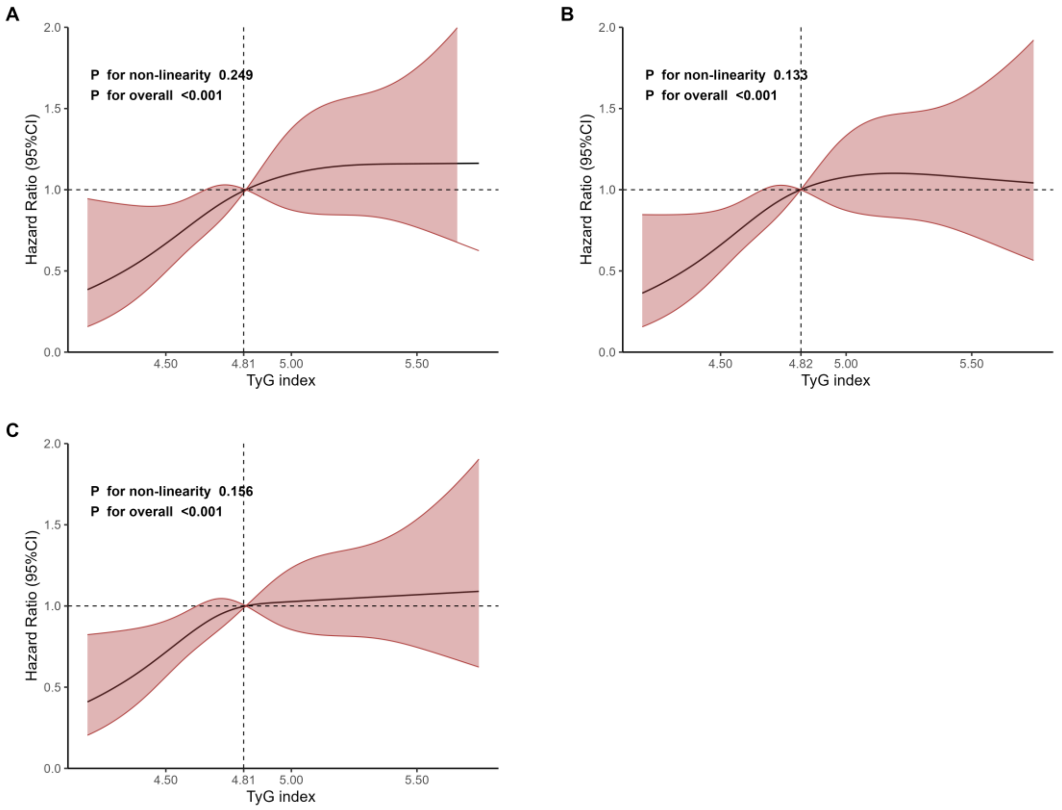

Fig. S6 Restricted cubic spline regression analyses of TyG index for mortality. **A** 90-day mortality **B** 180-day mortality **C** 5-year mortality

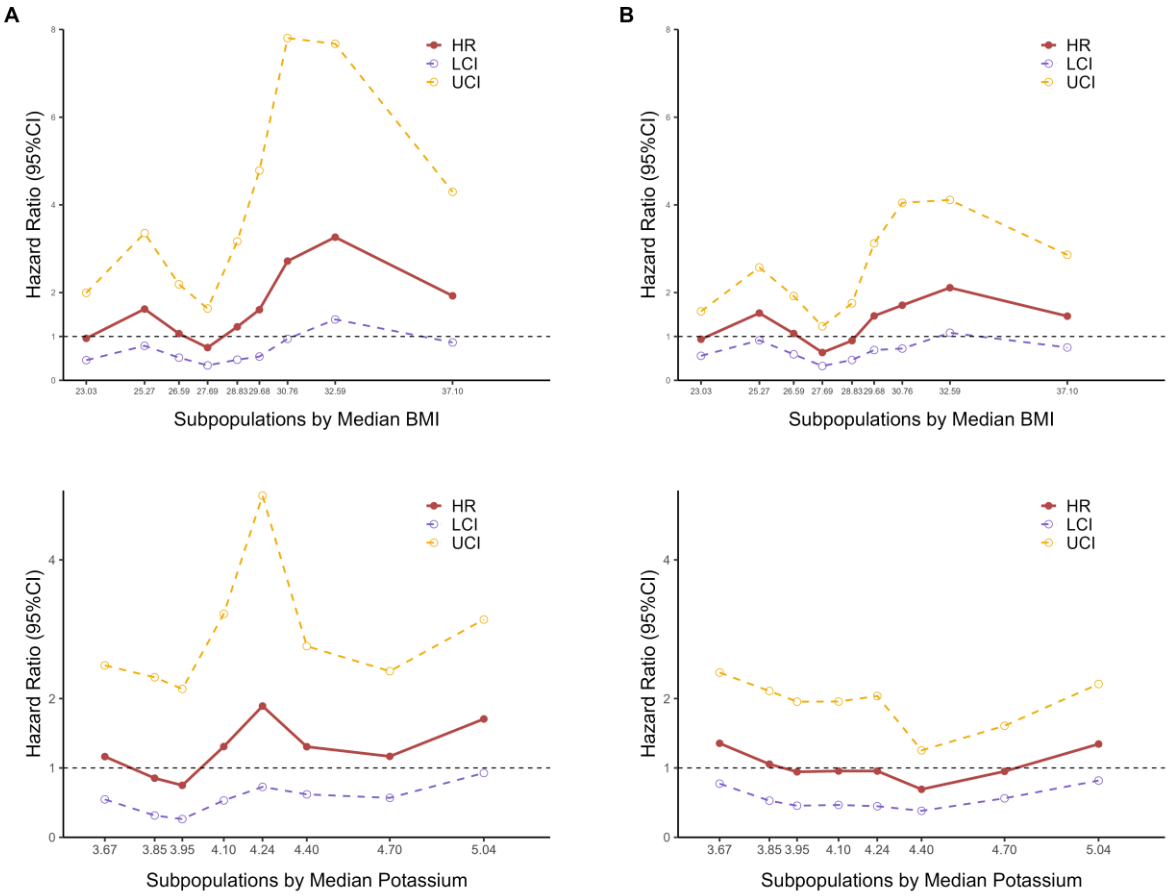

Fig. S7 STEPP analyses of mortality hazard ratios between two TyG index groups based on RCS nodes. **A** Subpopulations by median BMI for 28-day mortality. **B** Subpopulations by median BMI for 365-day mortality. **C** Subpopulations by median potassium levels for 28-day mortality. **D** Subpopulations by median potassium levels for 365-day mortality.

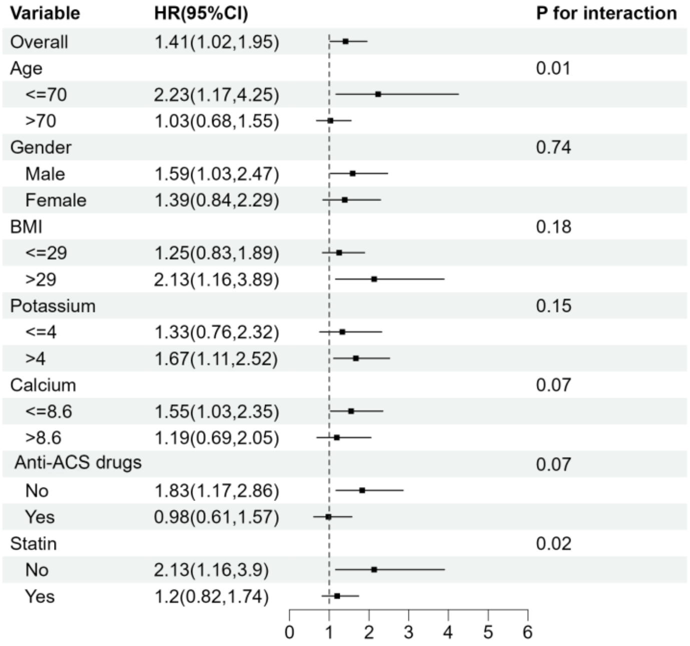

Fig. S8 Subgroup analyses for the association between TyG index and 90-day mortality

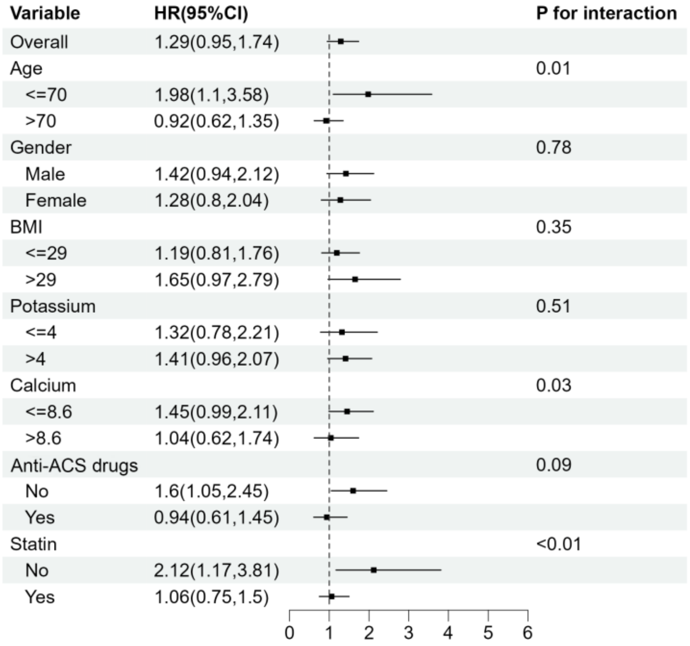

Fig. S9 Subgroup analyses for the association between TyG index and 180-day mortality

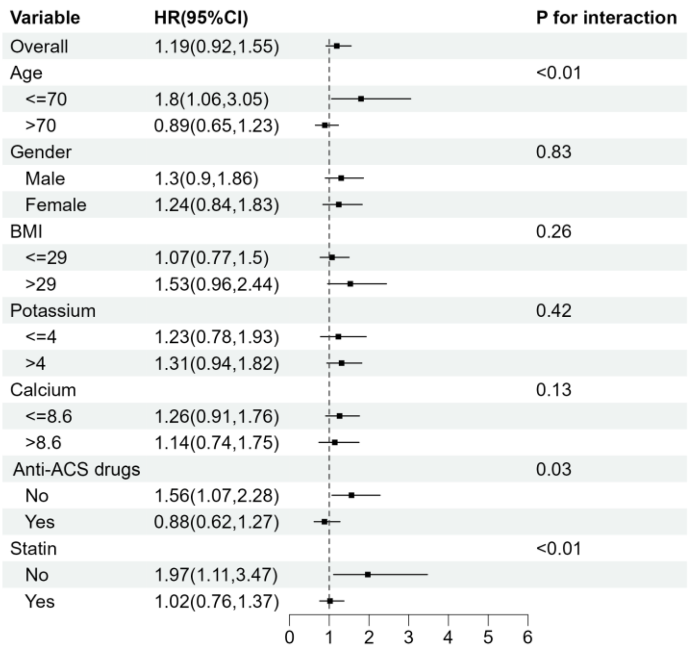

Fig. S10 Subgroup analyses for the association between TyG index and 5-year mortality

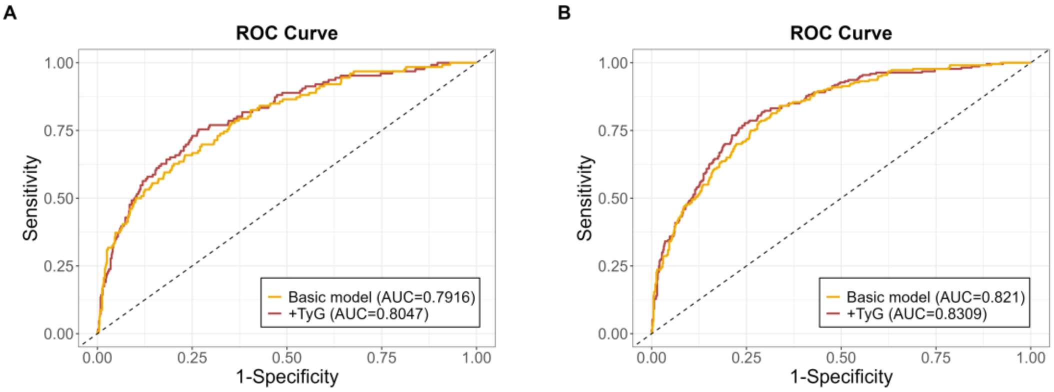

Fig. S11 ROC curve analyses of TyG index predicting mortality. **A** 28-day mortality **B** 365-day mortality

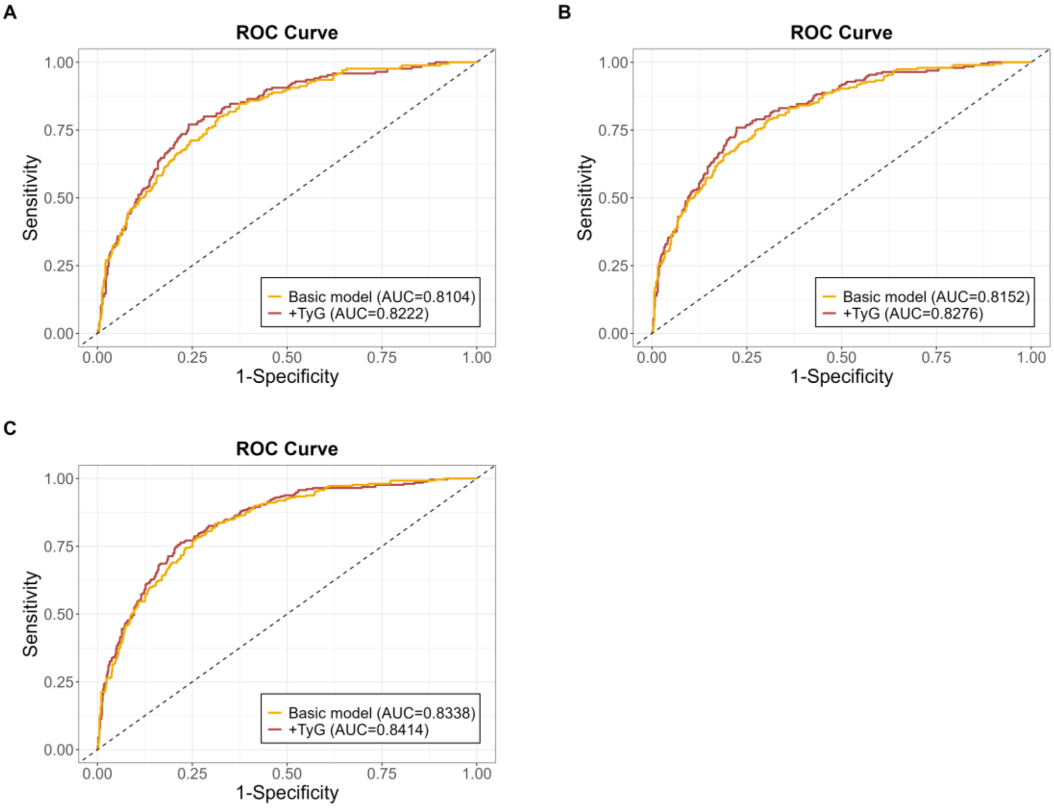

Fig. S12 ROC curve analyses of TyG index predicting mortality. **A** 90-day mortality **B** 180-day mortality **C** 5-year mortality
